# Annotations

**Published:** 1893-08-05

**Authors:** 


### ANNOTATIONS.

**Health Resorts: A Protest.**

The other day an evening newspaper reported that a plague of wasps had appeared close to a certain health resort on the South Coast, and that so numerous and excited had the insects been that several sheep had been stung in the throat and killed, and that other catastrophes might be apprehended. Now we would be the first to announce any real danger which might threaten visitors to any particular health resort, such, for example, as an epidemic of fever or the like; for we hold that the inhabitants of health resorts owe it to themselves and their patrons to make their towns and villages sanitarily perfect, simply because they are health resorts; and where they are too shortsighted and selfish to do this, we are glad to take a hand in their punishment by persuading people to stay away from their towns. But precisely because we do take such a stern view of the obligations of health resorts to protect their patrons against real dangers, are we all the more resolved not to raise the cry of "wolf" against any particular health resort when no wolf is in sight. There is a class of journalists—often mere boys—who think all is fish which comes to their net, and who have not the sense either to know or to care when they may be ruining deserving people. An outcry against the sanitation of a health resort may mean financial ruin to hundreds of heads of families for that year, and starvation to their children. Many struggling people are no doubt permanently tossed overboard into the deep sea of poverty every year by such senseless sensationalism. But what does the youthful journalist care? He has made his "copy" and his half guinea. We submit that editors should take a sterner and more honourable view of their responsibility. No man more bitterly resents injuries than the irritable man of the pen. Why cannot he take a leaf out of his own book of self-esteem, and rigidly abide by the rule of "doing as he would be done by?"

**Cholera Precautions in Villages.**

So far as ground and water sanitation are concerned, villages in this country are often in a much worse condition than large towns. Such water as can be taken out of running streams or "dipped" out of surface wells is used without hesitation for drinking purposes, and is ordinarily neither filtered nor boiled. But a moment's reflection shows that in thickly populated country districts the streams must be polluted by the soakage from innumerable farmyards on their banks and in their vicinity; and even the manure carted into the fields of the watershed must be largely washed down into the rills of the higher ground and the rivers of the valleys. That this is so, in fact, has been recently demonstrated in the west of Scotland by Dr. McVail, D.P.H., and others acting in conjunction with him. Dr. McVail has undertaken the investigation of the water supply of a large number of villages and small towns under the County Council of Stirling, in view of the threatened approach of cholera. By the authority of the County Council, Mr. Wilson, F.I.C., has analysed no fewer than seventy-four samples of water taken from different sources of supply in Stirlingshire. The results were alarming. "The great majority of the samples were found to be unfit for domestic use, and the whole facts were reported to the Committee." Now what is true of the county of Stirling and its villages is true of other counties and villages in varying degrees. Some streams and wells are purer than other streams and wells throughout Great Britain; but there is no doubt that if all our village sources of water supply were examined the reports of them would read exactly as the Stirlingshire report reads: "the great majority" of the samples of water analysed would be "found to be unfit for domestic use." The natural and proper thing to be done under these circumstances would be to provide a central and absolutely pure water supply, and to distribute the water to groups of villages and small towns by such methods as would render contamination in the transit impossible. But that at present must be looked upon as a counsel of perfection. What has been done in Stirlingshire is that each village has undertaken to protect the purity of its water by all available means. Pumps and dip wells have been repaired; the wells have been "puddled" to a certain depth to keep out the partially filtered surface water, and the streams are to be protected against the discharge into them of all possible impurities. The cost of all this has been very small. We would urge upon Medical Officers of Health and all small communities throughout the country the use of such means as Stirlingshire has adopted, until advancing civilisation shall provide central and abundant supplies of pure water. Since cholera is undoubtedly a "water-borne" disease, it is obvious that no single precaution can be so absolutely preventive as an abundant supply of perfectly pure water.

**Rich Deserters at West-end Churches.**

We look to the rich and the great for loyalty and devotion to great causes. Those who speak for "the classes" are never tired of telling us that the classes represent the traditions of nobility of conduct, of leadership, of conspicuous courage and resolution in the great world-battle. They are the natural object lessons to which the great multitude must constantly look, and whom they must imitate—*longo intervallo*. But this year, in the annual hospital battle for survival, the rich have in a great many instances taken to their heels and run away—sneaked, like cowards, clean out of the field. The West-end, with its bonnets and millinery and costly living has deserted the great cause. It is the poor who have been brave and true. Fie, ladies, of the West-end churches! This is not worthy of you. But a few years ago the "dear" slums claimed all the devotion of your hearts. To-day you—do not turn up your noses at the slums, for that would be vulgar—but you leave the slums entirely out of your consideration, and that is detestably vulgar, because it is detestably mean. The Hospital Sunday collections have fallen off by £5,000 this year, and the falling off has been entirely in the West-end churches. In the districts of London inhabited by the poor and the toiling there has been an actual increase in the contributions to the fund. But let us whisper to those Lady Falstaffs who have run away from duty that it is not too late for them to retrieve their honour. £5,000 will make up the deficit. Hasten, ye great ones of the West-end churches, and send to Sir Sydney Waterlow, not £5,000, but £10,000. Thus will you prove your penitence for the past and the loyalty of your purpose for the future. There is a special reason this year why the Hospital Sunday Fund should exceed rather than fall below its normal standard. Mr. J. D. Allcroft, the Chairman of St. Mary's Hospital, and a leading member of the Distribution Committee of the Sunday Fund, has died during the year. The other members of the committee would have been glad if such a memorial as a £50,000 collection could have been raised to his memory. As we have already said, it is not yet too late. We cannot afford to let the public and conspicuous good works of our great country fall to a low or mean standard, and so bring upon ourselves personal dissatisfaction and the loss of our world-wide reputation for good deeds.

